# Concordance between modification of diet in renal disease, chronic kidney disease epidemiology collaboration and Cockcroft-Gault equations in patients with chronic kidney disease at St. Paul’s hospital millennium medical college, Addis Ababa, Ethiopia

**DOI:** 10.1186/s12882-017-0783-3

**Published:** 2017-12-20

**Authors:** Hunduma Dinsa, Teshome Nedi, Alemseged Beyene Berha

**Affiliations:** 1 Department of Pharmacy, College of Medicine and Health Sciences, Ambo University, Ambo, Ethiopia; 2 Department of Pharmacology and Clinical Pharmacy, School of Pharmacy, College of Health Sciences, Addis Ababa University, P.O. Box 9086, Addis Ababa, Ethiopia

**Keywords:** CG, CKD, CKD-EPI, Drug dose adjustment, MDRD

## Abstract

**Background:**

The most commonly used glomerular filtration rate estimating equations for drug dosing are Cockcroft-Gault (CG), Modification of Diet in Renal Disease (MDRD), Chronic Kidney Disease Epidemiology Collaboration (CKD-EPI) equations. However there is still a concern about whether to use MDRD and CKD-EPI interchangeably with CG for drug dosage adjustment.

**Methods:**

The study was initiated to determine the concordance between MDRD, CKD-EPI and CG equations and associated factors in patients with chronic kidney disease at Saint Paul’s Hospital Millennium Medical College (SPHMMC). This was a cross sectional study which involved patient chart review and physicians self-administered questionnaire. Serum creatinine level ≥ 1.2 mg/dL was used as a cutoff point in pre-selection of patients. The correctness of the drug dose prescribed for the level of renal function were compared to the drug database (Lexi-Comp) available through Up-to-date version 21.2.

**Results:**

Among the total of 422 patients, 249 (59%) were males. Mean age of patients was 46.09 years. The use of MDRD equation for drug dose adjustment by physicians working in the renal clinic of SPHMMC was six out of nine physicians. The Pearson correlation coefficient between the CG with MDRD and CKD-EPI equations was *r* = 0.94, *P* < 0.001 and *r* = 0.95, *P* < 0.001, respectively. The concordance between the CG with MDRD and CKD-EPI equations for FDA assigned kidney function categories was 73.7%, Kappa = 0.644 and 74.9%, Kappa = 0.659, respectively. Concordance between the CG with MDRD and CKD-EPI equations for the drug dosing recommendation was 89.6%, kappa = 0.782 and 92%, kappa = 0.834, respectively. Age > 70 years was associated with discordance between CG and MDRD equations for drug dosing recommendation whereas serum creatinine 1.2–3.5 mg/dL, weight < 61 Kg and age > 70 years were associated with discordance between the CG with MDRD and CKD-EPI equations for FDA assigned kidney function categories. However, none of the factors associated with discordance between CG and CKD-EPI for drug dosing.

**Conclusion:**

MDRD equation can be used interchangeably with CG equation for drug dosing recommended in all adult patients between the age of 18 and 70 years. CKD-EPI can be used interchangeably with CG in all adult Ethiopian patients with CKD.

**Electronic supplementary material:**

The online version of this article (10.1186/s12882-017-0783-3) contains supplementary material, which is available to authorized users.

## Background

Chronic kidney disease (CKD) is the presence of kidney damage or a reduction in the glomerular filtration rate (GFR < 60 ml/min/1.73 m^2^) for three months or longer. It is a significant and widespread health problem with growing incidence and prevalence worldwide [1]. The estimated overall prevalence of CKD in adults is increasing exponentially in the older population [2]. The GFR estimating equations are affected by physiologic variables (age, race, sex, muscle mass, weight and height) of an individual. They have different qualities and significant limitations in the estimation of GFR [3]. Furthermore, they are not accurate in the population that are different from those in which the equations were developed [4]. In order to identify and manage CKD patients early, a more precise way of detecting kidney function and kidney injury in the clinical setting is essential [5]. On top of this, patients with CKD require appropriate medication dosing for disease severity and level of renal function for avoiding adverse drug events, preventing additional renal injury, and optimizing patient outcomes [6].

Hence, the most commonly used GFR estimating equations for drug dosing are the MDRD, CKD-EPI and CG eqs. [3, 7]. However, there is continued debate over which equation more accurately estimates renal function [8]. In accordance with current recommendation by Kidney Outcomes Quality Initiative (KDOQI) and National Institute for Health Excellence (NICE) [9, 10] are to use serum creatinine concentration to estimate GFR (eGFR) and transform it using the Chronic Kidney Disease Epidemiology Collaboration (CKD-EPI) eq. [11]. CKD-EPI replaces the Modification of Diet in Renal Disease (MDRD) eq. [12] as a more accurate predictor of clinical risk [13] and both these equations correct for selected non-renal influences (age, race, gender). To deliver better value through all changes in clinical practice by focusing on the clinical impact of standardization of creatinine measurement and implementation of eGFR reporting has created doubt and confusion among health care providers. A focus of concern is the assessment of kidney function for drug dosing adjustment [5].The great concern fear of overdosing or under dosing of the patient if the conventional use of CG in drug dosage adjustment is changed to the use of the MDRD and CKD-EPI [3, 14].

Numerous studies were done to compare these two equations for drug dosing recommendations in various settings. However, there is still no study done in Ethiopia, to compare the agreement between the MDRD and. CKD-EPI and CG equations for the drug dosing purpose. Since the mean weight of the population in which the MDRD equation was developed is equal to 80 Kg which is too high, the concordance may be different in Ethiopians. The aim of this study was to determine the concordance between MDRD and CKD-EPI and CG equations and associated factors in patients with CKD at St. Paul’s Hospital Millennium Medical College (SPHMMC).

## Methods

### Study area

The study was conducted in the renal clinic of SPHMMC, which is one of the referral hospitals in Addis Ababa under the Ethiopian Federal Ministry of Health (FMOH). On average 280 patients per month visit the renal clinic of SPHMMC.

### Study design

Hospital based cross-sectional survey was conducted from July, 2016 to September, 2016, through structured checklist which involved patient card review and structured questionnaires which was filled by physicians working in the renal clinic during the study period.

### Inclusion and exclusion criteria

All adult patients receiving at least one drug and have the most recent SCr ≥1.2 mg/dL were included. But pregnant women and patients with acute kidney injury were excluded as the CG; MDRD and CKD-EPI equations are not accurate in these patient groups.

### Sampling technique

All patients who visited the renal clinic of SPHMMC from July 2016 to September 2016 were considered for sampling purpose by using convenience sampling technique. To determine the prevalence of MDRD, CKD-EPI and CG use, all physicians working in renal clinic of SPHMMC during the study period were included.

### Data collection procedure

Weight and height of the individual patient were obtained by calibrated balance that was available in the renal clinic. For each patient in the study, eGFR was calculated using the CG, MDRD and the CKD-EPI equations; eGFR and eCrC were calculated by using the equations described below.

**eGFR using MDRD** = 186× (creatinine/88.4) −1.154 × age − 0.203× (0.742 if female) × (1.21 if black). Where, the result was expressed in ml/min/1.73 m^2^.

**eGFR using CKD-EPI** = 141 x min(SCr/κ, 1)^α^ x max(SCr /κ, 1)^-1.209^ × 0.993^Age^ × 1.018 [if female] ×1. 159 [if Black].Where, the eGFR = ml/min/1.73 m^2^; SCr (standardized serum creatinine) = mg/dL; κ = 0.7 (females) or 0.9 (males); α = −0.329 (females) or −0.411 (males); min = indicates the minimum of SCr/κ or 1; max = indicates the maximum of SCr/κ or 1; Age = years.

**eCrCl using CG** = [(140 − age) × weight in kg]/72× serum creatinine × (0.85 if female).

Where, the result was expressed in ml/min.

From among sampled patient cards which fulfilled the inclusion/exclusion criteria; those with eGFR lower than 60 ml per minute, according to CG and at least one drug prescribed were included for further analysis to determine concordance for drug dosing recommendation between the CG and the MDRD and CKD-EPI equations. After data collection had been completed, the correctness of the drug dose prescribed for the level of renal function were compared to the drug database (Lexi-Comp) available through Up-to-date version 21.2, which can be accessed by searching on any individual drug. To collect data from physicians, any physician working in the renal clinic during the study period filled only a single self-administered questionnaire on whether she/he has ever used these equations (Additional file 1).

### Ethical clearance

The study was conducted after ethical approval was obtained from the ethical review board of School of Pharmacy, Addis Ababa University (Ref.no PCP/449/08/16) and Institutional Review Board of SPHMMC (Ref.no PM23/294).Confidentiality of patient data was assured by recruiting data collectors from nurses working in the renal clinic. The name and address of the patient was not recorded on data abstraction format and only patient card number was used for identification.

### Data analysis

Data were summarized in percentage and presented in the form of tables and graphs. The odds ratio and 95% confidence interval were used to check significant association between dependent & independent variables using Bivariate and Multivariate analysis of logistic regression model. Concordance between the two equations for GFR agreement and drug dosing recommendation was tested using the kappa test. Pearson’s correlation coefficient was calculated to quantify the degree of linear relation between CG and MDRD equations. Bland-Altman analysis was used to show within person difference between the two equations. In all cases, *P*-value <0.05 was considered to be significant. All analyses were done using statistical package for social sciences (SPSS) version 20.

## Results

### Socio-demographic characteristics of study participants

**Fig. 1:**
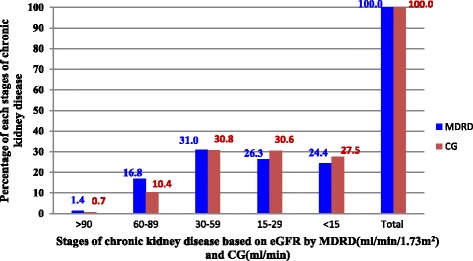
Frequency of chronic kidney disease stages among patients in renal clinic of St. Paul’s Hospital Millennium Medical College, Addis Ababa, Ethiopia between July – September, 2016 (*n* = 422)

From a total of 712 potential patients who visited the Renal Clinic of SPHMMC from July–September 2016, 422 patients were included in the study. As shown in Tables 1, 249 (59%) were males. Mean age of patients was 46.09 (SD 15.72). Of the total patients, 289(68.5%) have hypertension as co-morbidity followed by diabetes mellitus 115 (27.3%), and chronic glomerulonephritis 79 (18.7%). Of 422 CKD patients, 163 (38.6%) took at least one drug which needed dose adjustment for the level of renal function. Of these 163 patients, drug dose adjustment was incorrect for 59 (35.6%) according to MDRD equation and 69 (42.3%) according to CG equation. As Fig. 1 shows, CKD prevalence comparison between CG and MDRD equations, revealed a higher percentage (31.0% and 30.8%) of patients to the stage 3 CKD (eGFR 30–59) using CG and MDRD, respectively.

**Table 1:** Baseline characteristics of Patients with chronic kidney disease in renal clinic of St. Paul’s Hospital Millennium Medical College, Addis Ababa, Ethiopia between July – September, 2016 (*n* = 422)

Variables		N (%)
Age group (years)	<=30	88 (20.9)
	31–40	97 (23)
	41–50	78 (18.5)
	51–60	84 (19.9)
	61–70	47 (11.1)
	>70	28 (6.6)
Sex	Male	249 (59)
	Female	173 (41)
BMI(Kg/m2)	<18	35 (8.3)
	18–24.9	308 (73)
	25–30	67 (15.9)
	>30	12 (2.8)
Cause/Co-morbidity	HTN	177 (41.9)
	DM	17 (4.0)
	CGN	58 (13.7)
	Others	55((13)
	HTN,DM,CGN	1 (0.2)
	HTN,DM	94 (22.3)
	HTN,CGN	17 (4.0)
	DM,CGN	3 (0.7)
At least one drug need dose adjustment	Yes	163 (38.6)
	No	259 (61.4)
MDRD correctly adjusted	Yes	105 (64.4)
	No	58 (35.6)
CG correctly adjusted	Yes	94 (57.7)
	No	69 (42.3)

*HTN=Hypertension;DM = Diabetes Mellitus;CGN=Chronic Glomerulonephritis*

### Drug classes which need a dose adjustment among patients with chronic kidney disease and specific drugs

As shown in Table 2, the most frequent drugs which needed drug dose adjustment were cardiovascular drugs 88 (54%) followed by antibiotics 34 (20.9%) and anti-diabetics 24 (14.7%). As Table 3 shows there are certain specific drugs with disagreement between MDRD and CG equation for drug dose adjustment like metformin.

**Table 2:** Frequency of drug classes which need dose adjustment among Patients with chronic kidney disease in renal clinic of St. Paul’s Hospital Millennium Medical College, Addis Ababa, Ethiopia between July – September, 2016 (*n* = 163)

Drug Class	Frequency; N (%)
Cardiovascular drug	88 (54.0)
Antibiotics	34 (20.9)
Anti-gout	1 (0.6)
Drugs for bone disorder	1 (0.6)
Anti-diabetics	24 (14.7)
Histamine 2 blocker	2 (1.2)
ART drugs	10 (6.1)
Tramadol	2 (1.2)
Anti-lipidemics	1 (0.6)
Total	163 (100)

**Table 3:** Specific drugs with drug dose adjustment disagreement between CG and MDRD formula among patients with chronic kidney disease in renal clinic of St. Paul’s Hospital Millennium Medical College, Addis Ababa, Ethiopia between July – September, 2016 (*n* = 17)

Drug	Dosing guideline according to Up to date version 21.2	CG (ml/min)			MDRD (ml/min/1.73m^2^)		
		eCr Cl	Dose	Dose recommendation	eGFR	dose	Dose recommendation
Pyrazinamide 1200 mg tablet orally every 24 h	CrCl < 30 ml/min; 25–35 mg/kg three times per week	27	too high	Decrease	34	Normal	No change
Glyburide 2.5 mg tablet orally every 24 h	CrCl < 50 ml/min; contraindicated	31	CI	D/C	72	Normal	No change
		36	CI	D/C	67	Normal	No change
Metformin 500 mg tablet orally every 12 h	CrCl < 60 ml/min; Contraindicated(CI)	43	CI	D/C	72	Normal	No change
		59	CI	D/C	77	Normal	No change
		58	CI	D/C	62	Normal	No change
		55	CI	D/C	73	Normal	No change
		58	CI	D/C	77	Normal	No change
Metformin 500 mg tablet orally every 24 h		39	CI	D/C	69	Normal	No change
Trimethoprim-sulfamethoxazole 960 mg tablet orally every 24 h	CrCl 15–30 ml/min: 50% of recommended dose;CrCl < 15 ml/min:contraindicated	13	CI	D/C	18	too high	decrease by half
		21	too high	decrease by half	38	Normal	No change
		36	Normal	No change	30	too high	decrease by half
Trimethoprim-sulfamethoxazole 480 mg tablet orally every 24 h		24	Normal	No change	32	too low	double it
Ciprofloxacin 500 mg orally every 12 h	CrCl 30–50 ml/min: 250–500 mg orally every 12 h; CrCl 5–29 ml/min: contraindicated	20	too high	decrease interval to every 12 h	31	Normal	No change
		28	too high	decrease interval to every 12 h	31	Normal	No change
Atenolol 50 mg tablet orally every 24 h	CrCl 15–35 ml/min/1.73m^2^: maximum dose 50 mg every 24 h; CrCl <15 ml/min/1.73m^2^: maximum dose 25 mg every 24 h	15	Normal	No change	14	too high	decrease by half
		14	too high	decrease by half	19	Normal	No change

CI: contraindicated; D/C: discontinue; hrs: hours

### Prevalence of MDRD equation use by physicians

**Fig. 2:**
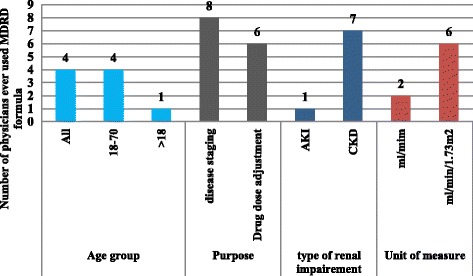
Prevalence of Modification of Diet in Renal Disease formula use by physicians in renal clinic of St. Paul’s Hospital Millennium Medical College, Addis Ababa, Ethiopia between July – September, 2016 (*n* = 9)

Of the 9 physicians, eight physicians used MDRD equation for CKD staging and six physicians used it for drug dose adjustment. Among eight physicians who used MDRD, six of them used BSA unmodified form of MDRD equation as shown Fig. 2.

### Correlation between eGFR by CG and MDRD and CKD-EPI equation

**Fig. 3:**
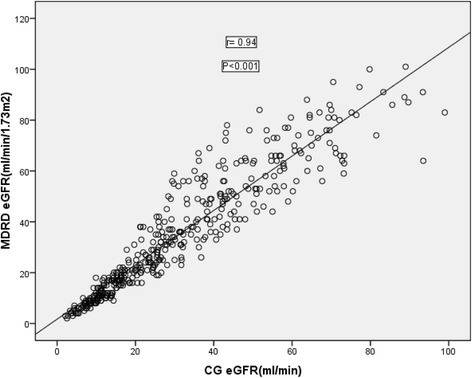
Correlation between estimated glomerular filtration rate by CG and MDRD in patients with chronic kidney disease in renal clinic of St. Paul’s Hospital Millennium Medical College, Addis Ababa, Ethiopia between July – September, 2016 (*n* = 422)

**Fig. 4:**
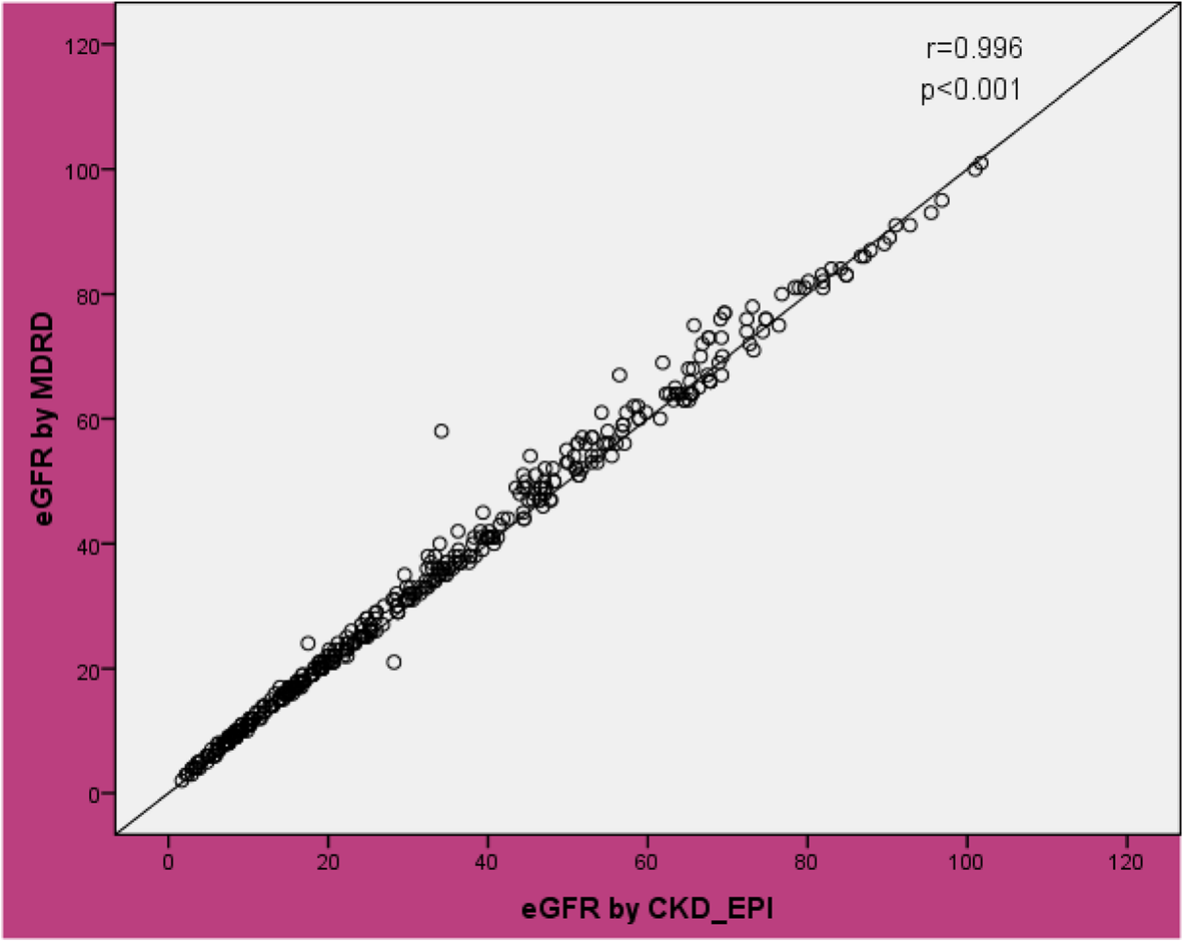
Correlation between estimated glomerular filtration rate by CKD_EPI and MDRD in patients with chronic kidney disease in renal clinic of St. Paul’s Hospital Millennium Medical College, Addis Ababa, Ethiopia between July – September, 2016 (*n* = 422)

As shown in Table 4, the CG equation correlated best with BSA modified MDRD equation than the BSA unmodified one. The r value was 0.94 with a *p* value of 0.001 for correlation between CG and BSA unmodified MDRD equation which can be seen in Fig. 3. However, the CG was correlated to the BSA modified MDRD equation with the r value of 0.97 and p value of 0.001.The correlation between CKD-EPI and CG was 0.946 (Table 4). As shown in Fig. 4, the correlation between CKD-EPI and MDRD was 0.996.

**Table 4:** Correlation between CG and MDRD and CKD-EPI formula in Patients with chronic kidney disease in renal clinic of St. Paul’s Hospital Millennium Medical College, Addis Ababa, Ethiopia between July – September, 2016 (*n* = 422)

Variables	Mean ± SD	R	*P*-value
MDRD BSA unmodified (ml/min/1.73m^2^)	34.76 ± 23.6	0.94	<0.001
CG (ml/min)	30.91 ± 20.69		
MDRD BSA modified (ml/min)	33.80 ± 24.06	0.97	<0.001
CG (ml/min)	30.91 ± 20.69		
CKD-EPI (ml/min/1.73m^2^)	33.34 ± 23.55	0.946	<0.001
CG (ml/min)	30.91 ± 20.69		
CKD-EPI (ml/min/1.73m^2^)	33.34 ± 23.55	0.996	<0.001
MDRD BSA unmodified (ml/min/1.73m^2^)	34.76 ± 23.6

### Concordance between CG and MDRD and CKD-EPI equation

As shown in Table 5, the concordance of the BSA unmodified MDRD Study equation with CG for FDA assigned kidney function categories was 73.7% compared to 80.3% for the BSA modified MDRD equation (*p* < 0.001). Concordance of BSA unmodified MDRD study equation with CG for recommended drug dosages was 89.6% as compared to 91.4% for BSA modified MDRD equation (*p* < 0.001). Likewise the concordance between CKD-EPI with CG and MDRD for GFR agreement was 92% and 94.5%, respectively.

**Table 5:** Concordance between CG and MDRD and CKD-EPI formula for GFR agreement and drug dosing recommendation in Patients with chronic kidney disease in renal clinic of St. Paul’s Hospital Millennium Medical College, Addis Ababa, Ethiopia between July – September, 2016 (*n* = 422)

		% of agreement	Kappa test	95% CI	*P*-value
For CKD staging	CG and BSA unmodified MDRD	73.7	0.644	0.58–0.70	.000
	CG and BSA modified MDRD	80.3	0.733	0.68–0.78	.000
	CG and CKD-EPI	74.9	0.659	0.60–0.72	.000
	CKD-EPI and unmodified MDRD	94.5	0.927	0.90–0.96	.000
For drug dose adjustment	CG and BSA unmodified MDRD	89.6	0.782	0.67–0.87	.000
	CG and BSA modified MDRD	91.4	0.820	0.73–0.91	.000
	Unmodified MDRD and CKD-EPI	94.5	0.881	0.81–0.96	.000
	CG and CKD-EPI	92	0.834	0.80–0.87	.000

**Fig. 5:**
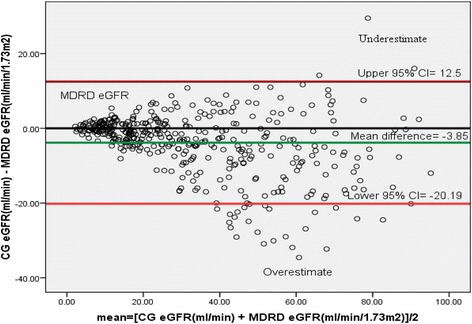
Bland-Altman plots between CG and MDRD formulas in patients with chronic kidney disease in renal clinic of St. Paul’s Hospital Millennium Medical College, Addis Ababa, Ethiopia between July – September, 2016 (*n* = 422)

**Fig. 6:**
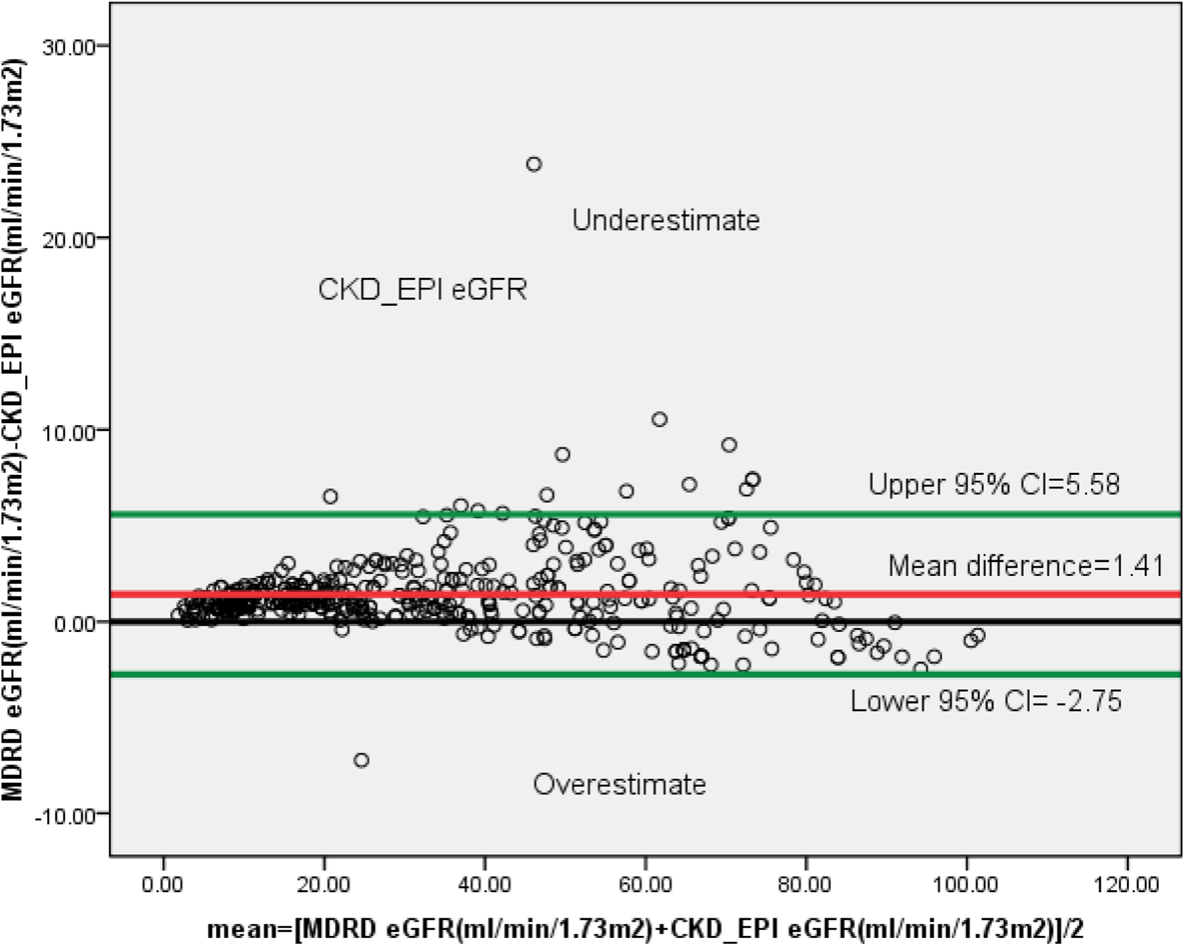
Bland-Altman plots between CKD_EPI and MDRD formulas in patients with chronic kidney disease in renal clinic of St. Paul’s Hospital Millennium Medical College, Addis Ababa, Ethiopia between July – September, 2016 (*n* = 422)

In our study, Bland-Altman analysis showed that within person differences between eGFR obtained by CG and MDRD. Agreement of the MDRD equation with CG was inspected visually by using Bland-Altman plots and quantified as the 95% limit of agreement between estimates. The limits of agreement represent a range of values within which the true difference between the two methods can be said to lie with 95% confidence. In the current study the true difference was between the lower 95% CI = −20.19 and upper 95% CI = 12.5 as shown in Fig. 5. MDRD equation overestimates eGFR at lower stages of CKD when compared to CG equation. Similarly Bland-Altman plot between eGFR by MDRD and CKD-EPI showed that the true difference was between the lower 95% CI = −2.75 and upper 95% CI = 5.58 as shown in Fig. 6.

### Factors associated with the concordance between CG and CKD-EPI equation for GFR agreement and drug dose recommendation

Weight less than or equal to 60 Kg, Age greater than seventy and serum creatinine greater than 3.5 md/dL were associated with discordance between CG and CKD-EPI equation for GFR agreement. However, there was no statistically significant difference between CKD-EPI and CG for the drug dose recommendation as shown in Table 6.

**Table 6:** Factors associated with the concordance between CG and CKD-EPI formula for GFR agreement in Patients with chronic kidney disease in renal clinic of St. Paul’s Hospital Millennium Medical College, Addis Ababa, Ethiopia between July – September, 2016 (*n* = 422)

Variables		GFR agreement		95% C.I.		COR	*P*-value	95% C.I.		AOR	*P*-value
		Yes	No	Lower	Upper			Lower	Upper		
Weight (Kg)	≤40	6	8			1					
	41–50	42	24	0.13	1.38	0.43	0.16	0.09	1.47	.369	0.157
	51–60	112	41	0.09	0.84	0.28	0.02	0.07	1.23	.283	0.091
	61–70	81	14	0.04	0.43	0.13	0.001	0.02	.50	.102	0.005
	>70	75	19	0.06	0.61	0.19	0.005	0.02	.61	.115	0.011
Age (years)	≤30	66	22			1					
	31–40	74	23	0.48	1.83	0.93	0.84	0.49	2.07	1.00	0.99
	41–50	59	19	0.48	1.96	0.97	0.92	0.51	2.36	1.10	0.80
	51–60	67	17	0.37	1.56	0.76	0.46	0.48	2.23	1.03	0.94
	61–70	37	10	0.35	1.90	0.81	0.63	0.39	2.43	0.97	0.94
	>70	13	15	1.43	8.39	3.46	0.006	1.87	13.25	4.98	0.001
Sr.Cr (mg/dl)	1.2–3.5	179	85			1					
	>3.5	137	21	0.19	0.55	0.32	0.000	0.14	0.45	0.25	0.000
BMI (Kg/m^2^)	<18.5	17	18			1					
	18.5–24.9	236	72	0.14	0.59	0.29	0.001	0.21	1.52	0.57	0.26
	25–30	55	12	0.08	0.51	0.22	0.001	0.18	2.57	0.68	0.57
	>30	8	4	0.12	1.86	0.47	0.28	0.17	6.56	1.05	.96

### Factors associated with concordance between CG and MDRD equation for GFR agreement and drug dose recommendation

As shown in Table 7 body weight from 61 to 70 Kg and greater than 70 Kg were associated with concordance between CG and MDRD equation for GFR agreement with 95% CI(0.032–0.803, *P* = 0.026) and 95% CI (0.020,0.621;*P* = 0.012) respectively. Similarly serum creatinine greater than 3.5 mg/dL is associated with higher concordance whereas age older than 70 years old is associated with discordance between the two equations with 95% CI (0.097, 0.327) and 95% CI (2.04, 15.33; *P* = 0.001) respectively. From Table 8, age older than 70 years is associated with the discrepancy between drug dosing recommendation based on the CG and MDRD equation with 95% CI (1.22,121.8;*P* = 0.034).

**Table 7:** Factors associated with the concordance between CG and MDRD formula for GFR agreement in Patients with chronic kidney disease in renal clinic of St. Paul’s Hospital Millennium Medical College, Addis Ababa, Ethiopia between July – September, 2016 (*n* = 422)

Variables		GFR agreement		95% C.I.		COR	*P*-value	95% C.I.		AOR	*P*-value
		Yes	No	Lower	Upper			Lower	Upper		
Weight (Kg)	≤40	6	8			1					
	41–50	39	27	0.16	1.67	0.52	0.27	0.12	2.15	0.51	0.36
	51–60	110	43	0.10	0.90	0.29	0.03	0.08	1.76	0.39	0.22
	61–70	78	17	0.05	0.53	0.16	0.003	0.03	0.80	0.16	0.03^a^
	>70	78	16	0.05	0.50	0.15	0.002	0.02	0.62	0.11	0.01^a^
Age (years)	≤30	66	22			1					
	31–40	77	20	0.39	1.55	0.78	0.48	0.39	1.78	0.84	0.64
	41–50	61	17	0.41	1.72	0.84	0.63	0.43	2.09	0.94	0.89
	51–60	60	24	0.61	2.36	1.20	0.60	0.93	4.23	1.99	0.08
	61–70	34	13	0.52	2.56	1.15	0.74	0.67	3.99	1.63	0.28
	>70	13	15	1.43	8.39	3.46	0.01	2.0	15.33	5.59	.001^a^
Sr.Cr (mg/dL)	1.2–3.5	172	92			1					
	>3.5	139	19	0.15	0.44	0.26	.000	0.10	.33	0.18	.000^a^
BMI (Kg/m^2^)	<18.5	15	20			1					
	18.5–24.9	230	78	0.12	0.52	0.25	.000	0.14	1.02	0.37	0.05
	25–30	58	9	0.04	0.31	0.12	.000	0.08	1.23	0.31	0.10
	>30	8	4	0.10	1.48	0.38	.16	0.13	5.27	0.82	0.83

^a^Statistically significant; COR: crude odds ratio; AOR: adjusted odds ratio; C.I. confidence interval

**Table 8:** Factors associated with the concordance between CG and MDRD formula for drug dosing recommendation agreement in Patients with chronic kidney disease in renal clinic of St. Paul’s Hospital Millennium Medical College, Addis Ababa, Ethiopia between July – September, 2016 (*n* = 163)

Variables		Drug dose recommendation agreement		95% C.I.		COR	*P*-value	95% C.I.		AOR	*P*-value
		Yes	No	Lower	Upper			Lower	Upper		
Sr.Cr (mg/dL)	1.2–3.5	83	15			1					
	>3.5	63	2	0.04	0.8	0.18	0.02	0.04	1.1	0.22	0.06
Age (years)	≤30	28	1			1					
	31–40	30	4	0.39	35.46	3.73	0.25	0.44	42.0	4.32	0.21
	41–50	25	1	0.07	18.86	1.12	0.94	0.07	19.3	1.13	0.93
	51–60	33	3	0.25	25.86	2.55	0.43	0.32	35.9	3.41	0.31
	61–70	22	3	0.37	39.28	3.82	0.26	0.33	36.6	3.49	0.30
	>70	8	5	1.78	172.17	17.50	0.01	1.22	121.8	12.17	0.03^a^

^a^Statistically significant; COR: crude odds ratio; AOR: adjusted odds ratio; C.I. confidence interval

## Discussion

The study was a cross sectional survey designed to assess the concordance between the MDRD and CKD-EPI and CG equations for CKD staging as well as for drug dosing recommendations in the renal clinic of SPHMMC. In this study, 422 patients were included in the determination of the correlation and concordance between the CG and MDRD and CKD-EPI equations in GFR agreement while only 163 (38.6%) patients were analyzed for determining concordance between these equations for drug dosing recommendation because the drug dose adjustment was required for the level of renal function only in 163 patients (Table 1).

The prevalence of MDRD use among physicians working in the renal clinic of SPHMMC was 8 out of nine physicians. There is limited study conducted on physicians’ use of MDRD equation, the current recommendation by KDOQI and NICE [9, 10] are to use serum creatinine concentration to eGFR and transform it using the CKD-EPI eq. [11] . CKD-EPI replaces the MDRD eq. [12] as a more accurate predictor of clinical risk [13] and both these equations correct for selected non-renal influences (age, race, gender).

The correlation between CG with BSA modified and BSA unmodified MDRD in this study was *r* = 0.97, *p* < 0.001 and *r* = 0.94, *P* < 0.001, respectively. This is similar with a study done by Gill et al.2007, in which the correlation between the CG and MDRD was very high that is *r* = 0.96 [14], but the result was greater than what was found by Căldăraru et al. 2011, where the Pearson correlation coefficient between the CG and MDRD equations was (0.83, *p* < 0.0001) which is lower than that of the current study because the former one used CG equation modified for body surface [15]. According to Melloni et al. 2008 correlation between CG and MDRD estimates of GFR was (*r* = 0.89; *p* < 0.0001) and it was conducted on 46,942 patients [8]. The Pearson’s correlation coefficient (95% confidence interval) was 0.84 (0.82–0.85) for CG versus MDRD [16]. This result is lower than our study because it was a community based cohort study, which did not restrict the serum creatinine of each participant. The Pearson’s correlation coefficient between the GFRs estimated by the CG and MDRD equations in the whole cohort was 0.828 (*p* < 0.001). This is still a lower score than our study because it was conducted without regard to the level of serum creatinine of each study participant [17]. Grillo et al. 2010, also found that correlation analysis of CG and MDRD estimates shown to be r^2^ = 0.69 (*p* < 0.0001) which is lower than the current study because the former one included patients’ serum creatinine between 0.8 mg/dL and 1.7 mg/dL [18].

The Spearman correlation coefficient between measured and estimated GFR for both equations was similar (4-V MDRD *r* ^*2*^ = 0.80 and CG *r* ^*2*^ = 0.79) but in our study the correlation was done between the MDRD and CG [19]. In the current study, very strong correlations were found between CKD-EPI and CG(*r* = 0.946, *P* < 0.001), CKD-EPI and MDRD(*r* = 0.996, *p* < 0.001) which were greater than what was found by Ruiz-Esteban et al**.** 2012 [20].

In the current study concordance between CG and BSA unmodified MDRD equation for FDA assigned kidney function categories were 73.7% compared to 80.3% for the BSA modified MDRD equation (*p* < 0.001). This result is lower than what was found by Steven et al. 2009, in which concordance between the MDRD study equation and the CG for GFR agreement was 78% (*p* < 0.001), probably because the latter one used BSA modified MDRD [21]. In the current study concordance between CG and MDRD equation for FDA assigned kidney function categories was compromised in underweight, aged population, i.e. older than 70 years and serum creatinine greater or equal to 1.2 mg/dL and less than 3.5 mg/dL. This result is similar in part with that of Roblin et al. 2009 in which most prominent differences were seen in aged and under-weighed subjects [22]. Park et al. 2012, also found that using MDRD in place of CG for dosage modification yielded higher dosing recommendations for subjects with a combination of age > 80 years, weight < 55 kg, and serum creatinine >0.7 and ≤1.5 mg/dL [23]. In addition, Eric et al. 2015, revealed that there is a difference between the results given by both MDRD and CG equations in the elderly over 65 years (*P* = 0.363) and in obese subjects (*P* = 0.142) [24]. However, in the current study obesity was not associated with discordance between CG and MDRD equation rather those patients having a body weight less than 61 Kg associated with discrepancy. Okparavero et al. 2013, also found that the concordance between CG and MDRD equation was 79%, kappa 0.69; 95 CI [0.60–0.77] which is greater than what was found in the current study [25]. Concordance between CG and MDRD for FDA assigned kidney function categories was weak k = 0.53, 95%CI [0.47,0.59] [16], as compared to the current study, which was moderate k = 0.644, 95% CI[0.58,0.70].This difference might be due to the fact that the current study was done on patients with renal impairment while the former study was community based. Park et al. 2012, found that the CG and the MDRD classification of renal function generally agreed (64.2%, κ = 0.54) [18]. MDRD-4 appeared to underestimate the fall in GFR with age compared with creatinine clearance (Ccr) using 24-h urine collections, CG and CKD-EPI which is similar to the current study in which the major discordance between the MDRD and CG equation was in older populations [26].

The concordance between CKD-EPI and CG for GFR agreement was 92%, while a study done by Delanaye et al. on patients age over 60 years was 45% [27]. CKD-EPI equation had higher concordance with CG as compared to MDRD for both GFR agreement and dosing recommendations which is similar with a study done by Okparavero et al. [25]. In our study factors associated with discordance between CKD-EPI and CG for GFR agreement were weight, age and serum creatinine (see Table 6) but in Delanaye et al. study age and weight had the strongest effect on the discrepancies [27]. Bland-Altman analysis was also carried out in the current study to check whether there was measurement agreement exist between CG and MDRD equation or not. As shown from Bland-Altman plots (see Fig. 5), MDRD equation had a strong measurement agreement with that of CG at lower eGFR and it became weaker at higher eGFR. MDRD equation overestimates eGFR at lower CKD stages compared to CG. This is similar to what was shown by other studies, in which MDRD overestimates eGFR at higher values [28, 29]. In stage 1–2, CKD eGFR overestimated measured GFR by 52.5, 38.0, and 19.3% for CG, MDRD (ethnicity-corrected), and MDRD (without correction), respectively, which is not similar to the current study in which MDRD equation overestimated eGFR at stage 1–2 CKD when compared to CG [30] . In our study Bland-Altman plot between eGFR by MDRD and CKD-EPI showed that the true difference was between the lower 95% CI = −2.75 and upper 95% CI = 5.58. The CKD-EPI equation underestimated eGFR at stage 2 as compared to MDRD equation as shown in Fig. 6.

In the present study, the concordance of BSA unmodified MDRD study equation with CG for recommended drug dosages was 89.6% as compared to 91.4% for BSA modified MDRD equation (*p* < 0.001). This result is similar with Steven et al. 2009, in which concordance rate between CG and MDRD study equation for recommended drug dosages 89% (*p* < 0.05) [21]. In the current study the discordance rate was 10.4% and it is similar to what was found by Manzano-Fernández et al. 2015 which was 10% [28]. Another study also found out the discordance rate between CG and MDRD for drug dosing recommendation to be 12.4% [29]. Golik et al. 2008 conducted a comparison of dosing recommendations for four antimicrobial drugs based on the CG and MDRD equations. They found discordance rate ranging from 22.8–36.3%, which is different from that of the current study because the former one included only patients’ serum creatinine with mean ± SD of 1.41 ± 0.95 mg/dL which contributes to the higher discordance [31].

According to Hermsen et al. 2009 study, level of concordance for the need for dosage adjustment based on the two equations was moderate (kappa coefficient 0.57, 95% confidence interval 0.5–0.63) [32] and their result is less than the current study, which is good (kappa 0.782, 95% CI 0.67–0.87.). The former study was exclusively done on antimicrobial drugs alone, whereas the current study included all drugs which needed drug dose adjustment for the level of renal function which might contribute to the difference between the two studies. In the current study, 82.4% of patients with discordant dose recommendation would receive a higher dose if the MDRD GFR was used while 99.1% of patients with discordant dose recommendations would receive a higher dose if the MDRD GFR was used in the study done by Hermsen et al. 2009 [32]. Another study also showed that in the 27 discordant cases, 67% of patients would have received a higher dose using the MDRD eq. [33]. In the current study, age > 70 years was associated with discordance between the two equations for the drug dosing recommendation. Since implementation of automatic reporting of MDRD calculated GFR in British Columbia, pharmacists have noted large discrepancies in drug doses calculated using MDRD and CG equations in elderly nursing home patients [14]. Another research, also found that those aged ≥80 years were more than 15-fold (OR = 15.41; 95% CI 10.00 to 23.75) more likely to be incorrectly dosed by MDRD [34]. For patients with advanced age further work is needed before the MDRD equations can replace the CG equation for dose adjustment in the labeling [23]. According to McFarland et al. 2011, the greatest discrepancy between CG and BSA modified MDRD for the drug dosing recommendation was observed in individuals over 75 years of age. This difference may be because, the latter study used BSA modified MDRD [35]. Another study has also shown that discrepancies between CG and MDRD derived drug dosing regimens have been observed in elderly patients [36]. All English-language articles were published before November 2007 searched on PubMed databases showed that to compare the use of this estimated GFR with estimated creatinine clearance (CrCl) calculated using the CG equation in the dosing of drugs requiring adjustments in elderly patients with declining renal function. None of the articles identified found that the use of the MDRD equation in the elderly was better than the CG for estimating renal drug elimination [37]. Our study also evaluated for the factors associated with discordance between CG and CKD-EPI for drug dosing purpose but there was no statistically significant difference between CKD-EPI and CG for the drug dose recommendation.

Limitations of the study are: Firstly, this studies evaluated, recommended doses of various renally excreted dugs rather than outcomes of different dosing with drug efficacy and safety. Study participants’ creatinine levels at the time of study visit were also assumed to be at the same levels at the time their drug dosages were determined. It was also better to compare the MDRD, CKD-EPI and CG equations with that of measured GFR. In addition, the sample size for physicians for assessing the prevalence of use of Modification of Diet in Renal Disease was too small.

## Conclusion

The concordance between CG and MDRD equations for drug dosing recommendation and for FDA assigned kidney function categories fall into good concordance that is concordance rate 89.6%, and 73.7%, respectively. Age older than 70 years associated with minimal concordance. Therefore; MDRD equation can be used interchangeably with CG equation for drug dosing recommendation in all adult Ethiopian patients between the age of 18 and 70 years. Similarly, the concordance between CG and CKD-EPI equations for drug dosing recommendation fall into very good concordance that is concordance rate 92%, kappa = 0.834 and for FDA assigned kidney function categories the concordance fall into good with concordance rate of 74.5%, kappa = 0.659. As there is no statistically significant discordance between the CG and CKD-EPI equation for drug dosing recommendation, the CKD-EPI equation can be used interchangeably in all adult Ethiopian patients with CKD for the drug dosing recommendation.

## Additional files

Additional file 1: Annex I. Questionnaires. Annex II: Data abstraction format. (DOCX 32 kb)

Additional file 2: Annex III-Consent Form. (DOCX 15 kb)

## Abbreviations

AOR: Adjusted Odds Ratio
BSA: Body Surface Area
CG: Cockcroft-Gault Equation
CKD: Chronic Kidney Disease
CKD_EPI: Chronic Kidney Disease Epidemiology Collaboration
COR: Crude Odds Ratio
CrCl: Creatinine Clearance
eGFR: Estimated Glomerular FiltrationRate
FDA: Food and Drug Administration
FMOH: Federal Ministry of Health
GFR: Glomerular Filtration Rate
KDOQI: Kidney Outcomes Quality Initiative
MDRD: Modification of Diet in Renal Disease
NICE: National Institute for Health Excellence
SCr: Serum Creatinine
SPHMMC: Saint Paul’s Hospital Millennium Medical College
