# Supplementary material for: Concordance between modification of diet in renal disease, chronic kidney disease epidemiology collaboration and Cockcroft-Gault equations in patients with chronic kidney disease at St. Paul’s hospital millennium medical college, Addis Ababa, Ethiopia
Source: BMC Nephrol. 2017 Dec 20;18:368. doi: 10.1186/s12882-017-0783-3 (PMC5738756; doi:10.1186/s12882-017-0783-3)
Supplement: Supplementary file 1 — Annex I. Questionnaires. Annex II: Data abstraction format. (DOCX 32 kb) [file 12882_2017_783_MOESM1_ESM.docx]

**Annex I. Questionnaires**

This questionnaire was used to collect data on the use of MDRD, Cockroft-Gault and other GFR estimating formula among physicians working in the nephrology clinic of SPHMMC during the study period

1. Demographic information

Age: ___________

Sex: _____________

Physician specialty:

Specialist

Resident

General practitioner

1. Have you ever used GFR estimating equation(s)?

Yes No If No, thank you.

1. If yes which one and for what purpose? You can choose more than one alternatives
2. Cockcroft-gault : For estimating GFR For drug dose adjustment For both
3. MDRD : For estimating GFR For drug dose adjustment For both
4. Others specify:
5. If your choice for question 3, is “B” and used it for drug dose adjustment, for which patient group?
6. Age>70 years
7. Age 18-70 years
8. Age<18 years
9. All age groups
10. Again if your choice for question 3, is “B” and used it for drug dose adjustment, for which type of renal impairment?
11. AKI
12. CKD
13. Which unit do you use for MDRD formula in drug dose adjustment?

A. ml/min B.ml/min/1.73 m^2^

# Annex II: Data abstraction format

This data abstraction format was used to collect data from CKD patient’s card at SPHMMC during the study period

1. Card No:_________
2. Sex: Male Female:
3. Age:_____years
4. Weight:________Kg
5. Height:_________cm
6. Date of hospital visit:_____________________________________________
7. Reason of Hospital visit:___________________________________________
8. Lab Findings:

| Scr (mg/dL) | BUN(mg/dL) | Date of measurement |
| --- | --- | --- |
|  |  |  |
|  |  |  |
|  |  |  |

1. List of drug prescribed during visit or Hospital stay

| S.N | Drug prescribed | Dose, frequency, administration, duration | Date prescribed |
| --- | --- | --- | --- |
| 1 |  |  |  |
| 2 |  |  |  |
| 3 |  |  |  |
| 4 |  |  |  |
| 5 |  |  |  |
| 6 |  |  |  |
| 7 |  |  |  |
| 8 |  |  |  |

1. Known Cause/Co morbid diseases:

1. Hypertension 2. Diabetes mellitus 3. Glomerulonephritis

4. Others specify: __________________
