# Supplementary material for: Concordance between modification of diet in renal disease, chronic kidney disease epidemiology collaboration and Cockcroft-Gault equations in patients with chronic kidney disease at St. Paul’s hospital millennium medical college, Addis Ababa, Ethiopia
Source: BMC Nephrol. 2017 Dec 20;18:368. doi: 10.1186/s12882-017-0783-3 (PMC5738756; doi:10.1186/s12882-017-0783-3)
Supplement: Supplementary file 2 — Annex III-Consent Form. (DOCX 15 kb) [file 12882_2017_783_MOESM2_ESM.docx]

# Annex III-Consent Form

Addis Ababa University

College of Health Science

Post Graduate Studies

School of Pharmacy

Department of Pharmacology and clinical pharmacy

Informed consent form prepared to compare MDRD equation with Cockcroft-Gault among patients who visit renal unit of St. Paul’s Hospital Millennium Medical College.

**Part I: Information about the Study**

My name is Hunduma Dinsa Ayeno; currently I am a graduate student at the department of

Pharmacology and Clinical Pharmacy, College of health science, Addis Ababa University. I am conducting a study entitled “Concordance between Modification of Diet in Renal Disease and Cockcroft-Gault formulas in chronic kidney disease patients at St. Paul’s Hospital Millennium Medical College”. If you participate in the study, it is important to know the prevalence of use of the MDRD formula for dosing recommendation. Furthermore, the result of this research is important to identify the degree of the agreement between the MDRD formula and Cockcroft-Gault formulas for drug dosing recommendations. The information which obtained from the study will also contribute for determining if Cockcroft-Gault equation might be replaced by the abbreviated MDRD equation for dosage adjustment in chronic kidney disease patients. I kindly request you to participate in this study by providing response for all the following questions. This information will be used only for research purpose. Your participation in this study is completely on voluntary bases and you have a right to refuse or interrupt the participation at any time. You will not write your name on the questionnaires. Your genuine participation is very important for the outcome of the research and all of your information is kept confidentially. If you need clarification you can ask questions .I would like to appreciate your participation.

Part II consent

Are you volunteer to participate in the study 1. Yes _______ 2.No__________

In signing this document, I am giving my informed consent to participate in the study entitled

“Concordance between Modification of Diet in Renal Disease and Cockcroft-Gault formulas in chronic kidney disease patients at St. Paul’s Hospital Millennium Medical College”. I have been informed that the purpose of this research. I have been informed that my participation in this study is willing full and voluntary even. I have right to refuse or interrupt from participating in the study any time and my name will not be mentioned on the questionnaire. I undersigned, have understood the purpose of the study and fully agreed to participate in the study.

Participant’s

Name ____________________ Signature __________ Date __________

Data Collector’s

Name ______________________ Signature _____________ Date ________

Supervisor’s

Name _________________________ Signature _____________ Date _____
